# High-Throughput Sequencing Reveals Bell Pepper Endornavirus Infection in Pepper (*Capsicum annum*) in Slovakia and Enables Its Further Molecular Characterization

**DOI:** 10.3390/plants9010041

**Published:** 2019-12-26

**Authors:** Jana Tomašechová, Richard Hančinský, Lukáš Predajňa, Ján Kraic, Daniel Mihálik, Katarína Šoltys, Silvia Vávrová, Miroslav Böhmer, Sead Sabanadzovic, Miroslav Glasa

**Affiliations:** 1 Biomedical Research Center of the Slovak Academy of Sciences, Institute of Virology, Dúbravská cesta 9, 84505 Bratislava, Slovak Republic; jana.tomasech@gmail.com (J.T.); Lukas.Predajna@savba.sk (L.P.); 2 Faculty of Natural Sciences, University of Ss. Cyril and Methodius, Nám. J. Herdu 2, 91701 Trnava, Slovak Republic; rhancinsky@gmail.com (R.H.); jan.kraic@ucm.sk (J.K.); daniel.mihalik@ucm.sk (D.M.); 3 National Agricultural and Food Centre, Research Institute of Plant Production, Bratislavská cesta 122, 92168 Piešťany, Slovak Republic; 4 Institute of High Mountain Biology, University of Žilina, Univerzitná 8215/1, 010 26 Žilina, Slovak Republic; 5 Comenius University Science Park, Comenius University in Bratislava, Ilkovičova 8, 841 04 Bratislava, Slovak Republic; katarina.soltys@gmail.com; 6 Department of Microbiology and Virology, Comenius University in Bratislava, Ilkovičova 6, 841 04 Bratislava, Slovak Republic; 7 Department of Molecular Biology, Comenius University in Bratislava, Ilkovičova 6, 841 04 Bratislava, Slovak Republic; vavrova.s@gmail.com (S.V.); bohmer6@uniba.sk (M.B.); 8 Department of Biochemistry, Molecular Biology, Entomology and Plant Pathology, Mississippi State University, Mississippi State, MS 39762, USA; SSabanadzovic@entomology.msstate.edu

**Keywords:** high-throughput sequencing, HTS, endornavirus, pepper, genome, genetic diversity

## Abstract

Ribosomal RNA-depleted total RNAs from a sweet pepper plant (*Capsicum annuum*, labelled as N65) grown in western Slovakia and showing severe virus-like symptoms (chlorosis, mottling and deformation of leaf lamina) were subjected to high-throughput sequencing (HTS) on an Illumina MiSeq platform. The de novo assembly of ca. 5.5 million reads, followed by mapping to the reference sequences, revealed the coinfection of pepper by several viruses; i.e., cucumber mosaic virus (CMV), watermelon mosaic virus (WMV), pepper cryptic virus 2 (PCV2) and bell pepper endornavirus (BPEV). A complete polyprotein-coding genomic sequence (14.6 kb) of BPEV isolate N65 was determined. A comparison of BPEV-N65 sequences with BPEV genomes available in GenBank showed 86.1% to 98.6% identity at the nucleotide level. The close phylogenetic relationship with isolates from India and China resulted in their distinct grouping compared to the other BPEV isolates. Further analysis has revealed the presence of BPEV in sweet or chili peppers obtained from various sources and locations in Slovakia (plants grown in gardens, greenhouse or retail shop). Additionally, the partial sequencing of two genomic portions from 15 BPEV isolates revealed that the Slovak isolates segregated into two molecular clusters, indicating a genetically distinct population (mean inter-group nucleotide divergence reaching 12.7% and 14.5%, respectively, based on the genomic region targeted). Due to the mix infections of BPEV-positive peppers by potato virus Y (PVY) and/or CMV, the potential role of individual viruses in the observed symptomatology could not be determined. This is the first evidence and characterization of BPEV from the central European region.

## 1. Introduction

Pepper (*Capsicum* sp.) is a vegetable crop of the Solanaceae family which is widely grown in temperate regions either for direct consumption or for the food and pharmaceutical industries. Both sweet and chili peppers are among the top ten most widely cultivated vegetables in the world [1,2]. However, pepper production can be often constrained by infections by viral pathogens, negatively affecting the yield or quality of the production [3]. On the other hand, persistent, nonpathogenic viruses belonging to several families are also found frequently in plants [4,5]. Bell pepper is not an exception to that, with a few partiti- and endornaviruses reported from the host; e.g., pepper cryptic viruses 1 and 2 (PCVs 1 and 2), bell pepper endornavirus (BPEV), hot pepper endornavirus (HPEV).

The *Endornaviridae* family, comprising the genera *Alphaendornavirus* and *Betaendornavirus*, includes viruses which have been identified in plants, fungi oomycetes and protists [6,7,8]. Members of these genera were reported from several important crops, such as rice, bean, barley, cucurbits, spinach or pepper [9]. Endornaviruses are characterized by a stable low copy number in their plant host, exhibiting no obvious symptoms or pathological effect on plants (with the exception of Vicia faba endornavirus) and efficient vertical transmission [8,10,11].

Bell pepper endornavirus (BPEV) belongs to the genus *Alphaendornavirus* [8]. The virus is characterized by a single linear single-stranded RNA genome of approximately 14 kb in length, containing a single open reading frame (ORF), which is translated into a large polyprotein. This polyprotein of ca. 4815–4884 aa contains several conserved functional domains, such as putative viral methyltransferase (MTR), helicase 1 (Hel-1), UDP-glycosyltransferase (UDG) and RNA-dependent RNA polymerase (RdRp) [10]. Endornaviruses seem not to form true virions and are usually found as dsRNA replicative intermediates [8,12].

In this work, the first complete genome of a European BPEV isolate from Slovakia (BPEV-N65) is reported, together with a partial molecular characterization of additional isolates from this region, contributing to the better understanding of the genetic complexity of this virus on a global scale.

## 2. Results

A total of 5,511,704 high-quality reads (with an average length of 135.5 bp) were obtained from the ribosomal RNA-depleted total RNAs of the N65 pepper sample using the Illumina MiSeq platform. Blast analyses of de novo assembled contigs (16,101 contigs with the length higher than 500 bp) indicated the presence of a complex infection, involving cucumber mosaic virus (CMV, genus *Cucumovirus*), watermelon mosaic virus (WMV, genus *Potyvirus*), pepper cryptic virus-2 (PCV2, genus *Deltapartitivirus*), and BPEV (genus *Alphaendornavirus*) (Table 1).

Two large contigs (8851 and 5874 bp in size) were initially identified by Blast analyses to match with BPEV sequences in the GenBank. Further mapping of sequence data on the publicly available complete BPEV genomes, performed with the Geneious software, resulted in the reconstruction of the nearly complete BPEV genome sequences, lacking only 24 nucleotides at the viral 3’ terminus, as compared to NC_039216.

The coding complete sequence of the Slovak isolate BPEV-N65 was determined to be 14,655 nucleotides (nt) in length, putatively encoding a large polyprotein of 4884 aa (552.9 kDa). Like other endornaviruses, the polyprotein contains several conserved domains as determined by Conserved domain database (CDD) search; i.e., putative viral methyltransferase (MTR; aa 327–562), RNA helicase 1 (Hel-1; aa 1413–1649), glycosyltransferase (GT; aa 3113–3455) and RNA-dependent RNA polymerase (RdRp; aa 4446–4784). No additional conserved functional domains could be identified by searching in the CDD database. A comparison of the genome sequences revealed that the Slovak N65 isolate shares sequence identity with the other 15 BPEV isolates, ranging from 86.1% to 98.6%. at the nucleotide level. The deduced length of polyprotein in BPEV isolates was either 4884 aa (most of isolates) or 4815 aa (AB597230, JN019858, KU923755, KU923756, KP455654, MG545489, KT149366) depending on the AUG codon considered as an initiation codon for genome translation. The identity at the amino acid level of N65 with other isolates reached 89.8% to 98.9%.

**Figure 1:**
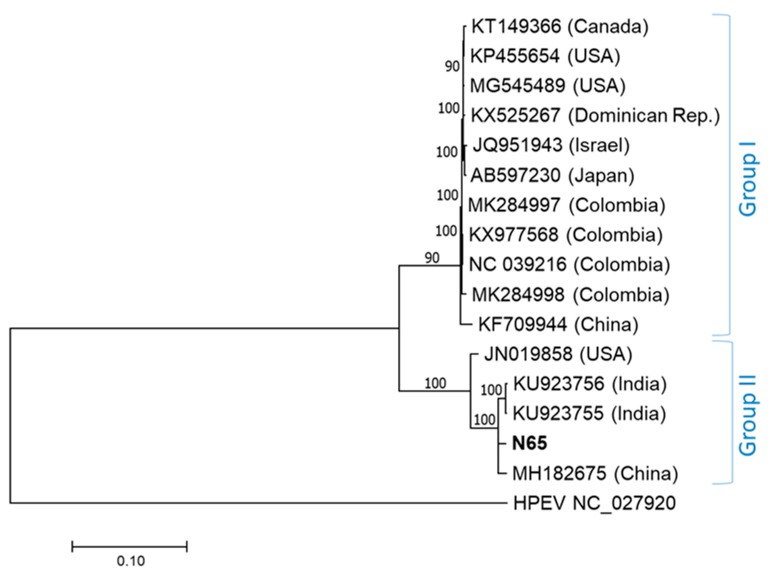
Phylogenetic tree generated on complete nucleotide genome sequences of bell pepper endornavirus (BPEV) isolates. The phylogenetic analysis was inferred using maximum likelihood (ML) based on the General Time Reversible (GTR) model selected as the best-fit model of nucleotide substitution based on Bayesian information criterion (BIC) as implemented in MEGA 7. Isolates are identified by their GenBank accession numbers and country of origin. The Slovak isolate N65 sequenced in the present study is highlighted in bold. Hot pepper endornavirus (HPEV) was used as an outgroup. The scale bar indicates a genetic distance of 0.1. Bootstrap values higher than 70% (1000 bootstrap resamplings) are indicated.

Phylogenetic analysis based on the full-length genome sequences showed the division of currently characterized BPEV isolates into two genetic groups (Figure 1). The isolate BPEV-N65 resulted as most closely related to BPEV isolates from China (MH182675) and India (KU923755, KU923756). These four isolates, along with an exemplar isolate “BPEV-YW” from USA (JN019858), form a highly supported cluster representing one of the two distinct evolutionary lineages of members of this species. A similar phylogenetic topology was obtained by the analysis of the polyprotein amino acid sequences, further supporting the phylogenetic affinity of BPEV-N65 with isolates from the Far East and from the USA.

To investigate further the occurrence and molecular diversity of BPEV in Slovakia, leaf samples were obtained during July–August 2018 from randomly selected pepper plants grown in private gardens in Piešťany (*n* = 10) and Pezinok (*n* = 10), in an insect-proof greenhouse in Bratislava (*n* = 5), and in May 2019 from a large number of young pepper plants in a retail shop in Bratislava (*n* = 5) (all locations in western Slovakia).

Primers targeting two different parts of the BPEV genome (nts 7776–8485 and 12,632–13,238) were designed based on the conserved regions among publicly available BPEV sequences, including HTS-generated data of isolate N65. From a total of 30 samples analyzed by reverse transcription polymerase chain reaction (RT-PCR), 15 samples tested positive for BPEV using both primer sets. The specificity of RT-PCR was checked by the direct sequencing of PCR products, resulting in the partial sequences of 669 and 567 bp, after primer removal, of 15 additional BPEV isolates from Slovakia.

**Figure 2:**
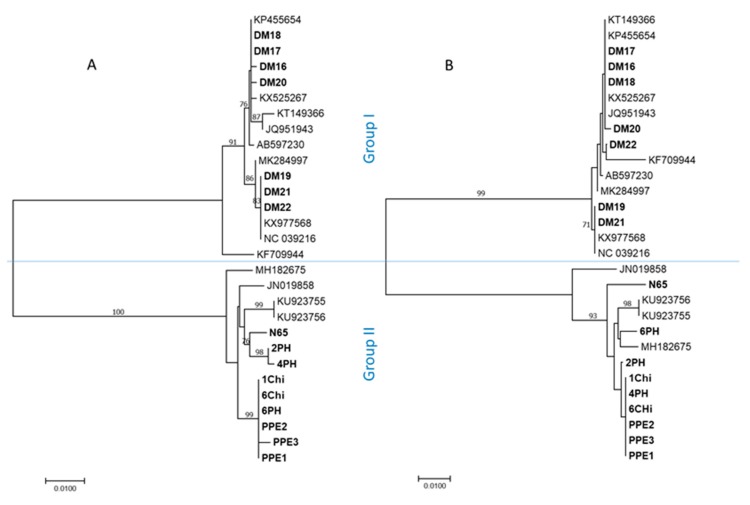
Neighbor-joining phylogenetic tree constructed from alignments of partial bell pepper endornavirus (BPEV) sequences of the genomic regions encompassing nts 7797–8465 (**A**), and nts 12,652–13,218 (**B**) based on the reference NC_039216. Slovak BPEV isolates are in bold. The sequences of previously characterized isolates are identified by their accession numbers and geographical location. Only bootstrap values ≥70% (1000 bootstrap resamplings) are indicated. The scale bar indicates a genetic distance of 0.01.

Phylogenetic analyses of both genome portions resulted in congruent tree topology, separating the studied Slovak isolates into two distinct phylogenetic groups (Figure 1, Figure 2A,B). Seven Slovak BPEV isolates from Piešťany fall into the major group I. Remaining isolates from Bratislava and Pezinok were grouped together with N65 in the group II. While the intra-group nt diversity among Slovak BPEV isolates remained low (0.2% and 0.3%, respectively), the mean inter-group divergence reached 12.7% and 14.5%, respectively, based on the genome portion analyzed.

## 3. Discussion

The persistent phytoviruses, also known as “cryptic” because of lack of obvious symptoms associated with their presence in plant hosts, have been poorly studied in the past. The use of unbiased HTS technologies for the study of the plant virome revealed their frequent occurrence in cultivated and non-cultivated plants [4,13]. The members of the *Endornaviridae* family have been reported in several economically important crops; however, their effect on the host phenotype is still poorly understood [14,15]. Furthermore, the extent of their genetic variability is still not fully understood due to the paucity of data from some geographical areas.

To date, the genomes of 15 BPEV isolates have been completely sequenced. However, those reports come only from Asian or American continents. To supplement the data on BPEV genetic diversity in the European region, the complete genome of a Slovak BPEV isolate from a sweet pepper plant, referred to as N65, was determined along with partial sequence data from an additional 15 BPEV isolates from sweet to chili pepper.

The analysis of a limited number of pepper samples from different locations in Slovakia (*n* = 30) showed BPEV infections in half of tested plants. Based on the phylogenetic analyses and pairwise comparisons, the studied Slovak BPEV isolates are not genetically uniform and belong to two previously defined molecular groups [5] (referred here as groups I and II). Although the number of BPEV sequences is still limited, the phylogenetic grouping (Figure 1 and Figure 2A,B) of sequenced isolates indicates an absence of geography-based clustering on a global scale. Accordingly, the separation of partially sequenced isolates from Slovakia into two groups is most likely due to different host genotype (variety) sampled rather than geography-driven divergence. Indeed, the co-evolution of endornaviruses with a specific type of plant genotype, as a consequence of a long coexistence of host and a virus, has been hypothesized recently for Cucumis melo endornavirus (CmEV; [16]), as well as for BPEV [9,10]. Accordingly, in this study, four BPEV isolates from chili peppers were grouped in the same phylogroup, suggesting adaptation and coevolution with a particular host genotype. The two primer pairs, designed on the database and HTS-based sequence determined in this work, were efficiently used in RT-PCR for the amplification of both genetic groups of isolates in the two portions targeted (spanning nts 7776–8485 and 12,632–13,238), thus showing a broad polyvalence to known BPEV variants.

A recombination has been shown to occur within the BPEV population [5,9]. Our recombination detection program (RDP) analysis of the dataset of 16 available complete BPEV sequences revealed one recombinant isolate (JN019858) showing a mosaic recombination pattern, involving the following:

- The nt coding region 4832–5542 (major parent N65, minor parent KX977568, overall probability 2.9*e^−83^, supported by 7/7 programs);
- The nt coding region 6320–7134 (major parent MH182675, minor parent KX977568, overall probability 1.3*e^−91^, supported by 7/7 programs).

Interestingly, the recombination events reported for the isolate “Penol” (NC_039216) in previous work [5] could not be confirmed in our study. It is possible that other recombination event(s) could be found if more genetically different variants of BPEV are available.

The phylogenetic analysis of Slovak isolates targeting two different genome portions did not show an incongruence in their affiliation to the respective genetic groups, which is consistent with an absence of recombination, at least in the nt 7776–13,238 region.

HTS technologies provide an enormous volume of sequence data, enabling many possible applications, including the identification of the virome, full-length genome characterization of known or emerging viruses in infected plants, or pathogen characterization without a priori knowledge [17]. Indeed, in this work, the complex virome has been identified in a symptomatic pepper plant, comprising members from the genera *Cucumovirus*, *Potyvirus*, *Deltapartitivirus* and *Alphaendornavirus*. This finding further emphasizes the intricate nature of plant viral diseases [18], indicating that the occurrence of complex viral infections in plants is rather a rule than an exception.

Different templates used for library preparation and different strategies for the enriching of viral sequences can be applied prior to the HTS analysis; e.g., virus-derived small interfering RNAs, double-stranded RNAs, ribosomal RNA-depleted total RNAs and virion-associated nucleic acids [19,20,21]. Our results confirmed the suitability of the total RNA templates, in which the virus fraction was enriched by ribosomal RNA depletion prior to library preparation for HTS. The HTS analysis of the pepper N65 sample enabled us to obtain complete or nearly complete genome sequences of several plant viruses, including acute pathogens (CMV, WMV) or persistent viruses (BPEV, PCV2).

Although the endornaviruses are considered as non-pathogenic, and their infection to be associated with no visible effect on their host, their potential contribution to plant fitness is not completely elucidated. In our work, due to the detection of the additional mixed infection of peppers by potato virus Y and/or cucumber mosaic virus, the potential role of individual viruses in the observed symptomatology (Table 2) could not be determined.

## 4. Materials and Methods

### 4.1. Analysis of the Virome and Determination of the BPEV Full-Length Genome Sequence by HTS

Sweet pepper plant (*Capsisum annum*, cv. Promotor), grown in a private garden in Čachtice, western Slovakia (GPS coordinates 48°42’38.2” N, 17°47’23.2” E) and showing leaf chlorosis, mottling and deformation symptoms, was sampled in August 2017.

Total RNAs were extracted from upper leaves of pepper plants using the Spectrum Plant Total RNA Kit (Sigma Aldrich, St. Louis, MO, USA). Ribosomal RNA was removed using the Ribo-Zero rRNA Removal Kit (Illumina, San Diego, CA, USA). The sample of ribosomal RNA-depleted total RNA was used for double stranded cDNA synthesis using the SuperScript II kit (Thermo Fisher Scientific, Waltham, MA, USA). The cDNA was then purified with the 2.2 x AMPure XP beads and quantified with the Qubit 2.0 Fluorometer (Thermo Fisher Scientific, Waltham, MA, USA). Subsequently, the sample was processed with the transposon-based chemistry library preparation kit (Nextera XT, Illumina, San Diego, CA, USA). Low-cycle PCR and mutual indexing of the fragments was carried out. Fragments were purified with 1.8 x AMPure XP beads (Beckman Coulter, Brea, CA, USA) without size selection. The fragment size structure of the DNA library was assessed using the Agilent 2100 Bioanalyzer (Agilent Technologies, Santa Clara, CA, USA). Since the obtained fragment length distribution (150–700 bp) met the criteria for further sample processing, the average bp value was used for sample molarity calculation. The equimolar pool of 4 nM DNA libraries was denatured, diluted to 10 pM and sequenced (300 bp paired-end sequencing) on the Illumina MiSeq platform (Illumina, San Diego, CA, USA).

High-quality trimmed reads were used for de novo assembly and contigs aligned to the viral genomes database [22] using Geneious v.8.1.9 software. Alternatively, the reads were mapped against the selected full-length sequences of viruses identified in the previous step to map the de novo assembled contigs. Alternatively, the reads were mapped against the full-length sequences of BPEV (retrieved from Genbank). Sanger sequencing of PCR products obtained by using specific primers designed from the HTS-based sequence was used to validate approximately a third of genome.

### 4.2. RT-PCR Detection and Partial Molecular Characterisation of Additional BPEV Isolates

Leaf samples were obtained during vegetation periods of 2018 and 2019 from sweet and chili pepper (*Capsicum annuum* L.) plants growing in private gardens, greenhouse or commercial retail shop in western Slovakia.

Total RNAs were extracted from pepper leaves using the NucleoSpin RNA Plant kit (Macherey-Nagel, Duren, Germany), and a two-step protocol was systematically used. The first-strand cDNA was synthesized using random hexamer primers and the AMV reverse transcriptase (Promega Corp., Madison, WI, USA). An aliquot of the RT reaction was analyzed via PCR carried out with TaKaRa Ex Taq polymerase (TaKaRa, Bio Inc., Shiga, Japan). Two distinct genome regions were amplified using primer pair dBPEV_12632F (5´ATGACCAAYGGGCAAGTACC3´, forward)/dBPEV_13238R (5´CTGTATCTTCCCAGAGACTC3´, reverse) and BPEV_7776F (5´AACCAGCAGAAACAAGCAAAG3´, forward)/BPEV_8485R (5´GGTGTATGCTACTGTGTGAC3´, reverse), designed on aligned sequences of N65 and all BPEV isolates are available in the GenBank database (accessed on June 2018).

The RT-PCR products were gel-purified using the Wizard SV Gel and PCR Clean-Up System (Promega Corp., Madison, WI, USA), and directly sequenced in both directions by priming the sequencing reactions with the same oligonucleotides used for PCR amplification.

The presence of PVY and CMV in the samples was detected by RT-PCR as previously described [23].

### 4.3. Sequence Analysis

The complete genome sequence of the Slovak BPEV isolate was compared with those of 15 BPEV isolates available in the GenBank database [24] (accessed on June 2018, see Figure 1 for their accession numbers). Sequence analyses were performed using either MEGA v.7 [25] or DnaSP [26]. Protein domain and motif searches were performed using the Conserved Domain Database (CDD) [27]. The phylogenetic trees were inferred using the maximum likelihood and neighbor-joining algorithm implemented in MEGA v.7. The bootstrap analysis with 1000 replicates was performed to assess the robustness of the branches. Searches for potential recombination events employed seven methods, including RDP, GENECONV, Bootscan, Maxchi, Chimaera, SiScan and 3SEQ, implemented in the RDP4 v.4.1 software [28].

The complete and partial nucleotide BPEV sequences reported in this paper have been deposited in the GenBank database under the accession numbers listed in Table 2.

## Figures and Tables

**Table 1:** Virome of the pepper N65 HTS dataset as resulted by mapping of 5,511,704 high-quality reads with Geneious software.

Virus Acronym and Genome Segment	Reference Genome Sequence	Reads Mapped against Reference	Percentage of Genome Covered	Coverage Depth
BPEV *	NC_039216	2535	99.8%	23.2 x
CMV RNA1	NC_002034	2498	100%	97.9 x
CMV RNA2	NC_002035	1868	100%	83.9 x
CMV RNA3	NC_001440	3770	100%	207.6 x
WMV *	NC_006262	411	93.0%	6.3 x
PCV2 RNA1	NC_034159	3378	98.3%	300.3 x
PCV2 RNA2	NC_034167	5514	99.7%	523.5 x

Legend: BPEV—bell pepper endornavirus; CMV—cucumber mosaic virus; WMV—watermelon mosaic virus; PCV2—pepper cryptic virus 2. *—genome composed of single ssRNA molecule.

**Table 2:** List of pepper samples and their characteristics. PVY: potato virus Y.

Sample	Locality	Type of Plantation/Pepper Type/Year of Sampling	Symptoms Observed	Detection of Additional Viruses		BPEV GenBank Accession Numbers
				PVY	CMV	
N65	Čachtice	Garden/sweet pepper cv. Promotor/2017	M, Mot, De	−	+	MN580384 ^a^
DM16	Piešťany	Garden/sweet pepper/2018	M	−	−	MN580386 ^b^, MN580405 ^c^
DM17	Piešťany	Garden/sweet pepper/2018	0	−	+	MN580390 ^b^, MN580406 ^c^
DM18	Piešťany	Garden/sweet pepper/2018	M	−	-	MN580385 ^b^, MN580407 ^c^
DM19	Piešťany	Garden/sweet pepper/2018	M, De	−	−	MN580387 ^b^, MN580408 ^c^
DM20	Piešťany	Garden/sweet pepper/2018	Mot	−	+	MN580389 ^b^, MN580409 ^c^
DM21	Piešťany	Garden/sweet pepper/2018	Mot	−	+	MN580388 ^b^, MN580410 ^c^
DM22	Piešťany	Garden/sweet pepper cv. Anka/2018	0	−	−	MN580391 ^b^, MN580411 ^c^
PPE1	Pezinok	Garden/sweet pepper cv. Slovakia/2019	De	−	−	MN580399 ^b^, MN580413 ^c^
PPE2	Pezinok	Garden/sweet pepper cv. Slovakia/2019	0	−	−	MN580397 ^b^, MN580412 ^c^
PPE3	Pezinok	Garden/sweet pepper cv. Slovakia/2019	M	-	+	MN580398 ^b^, MN580414 ^c^
2PH	Bratislava	Retail shop/chili pepper/2019	0	−	+	MN580393 ^b^, MN580401 ^c^
4PH	Bratislava	Retail shop/chili pepper/2019	M	+	−	MN580395 ^b^, MN580402 ^c^
6PH	Bratislava	Retail shop/sweet pepper/2019	M	+	-	MN580392 ^b^, MN580404 ^c^
1Chi	Bratislava	Greenhouse/chili pepper/2018	M, Mot, De		+	MN580394 ^b^, MN580400 ^c^
6Chi	Bratislava	Greenhouse/chili pepper cv. Habanero Lemon/2018	M, Mot, De	+	+	MN580396 ^b^, MN580403 ^c^

^a^/ complete genome, ^b^/ nts 7797–8465, ^c^/ nts 12,652–13,218. 0—symptomless, M—mosaics, Mot—mottling, De—leaf deformation.
